# Randomized, Phase II study of pemetrexed plus bevacizumab versus pemetrexed alone after treatment with cisplatin, pemetrexed, and bevacizumab in advanced non‐squamous, non‐small cell lung cancer: TORG (thoracic oncology research group) 1321

**DOI:** 10.1002/cam4.6135

**Published:** 2023-05-24

**Authors:** Takashi Kasai, Kiyoshi Mori, Yoichi Nakamura, Nobuhiko Seki, Yasuko Ichikawa, Haruhiro Saito, Tetsuro Kondo, Kazuo Nishikawa, Satoshi Otsu, Akihiro Bessho, Hiroshi Tanaka, Hiroyuki Yamaguchi, Takayuki Kaburagi, Hisao Imai, Keita Mori, Junya Ohtake, Hiroaki Okamoto

**Affiliations:** ^1^ Division of Thoracic Oncology, Department of Medical Oncology Tochigi Cancer Center Utsunomiya Japan; ^2^ Division of Thoracic Oncology, Department of Thoracic Diseases Utsunomiya Memorial Hospital Utsunomiya Japan; ^3^ Division of Medical Oncology, Department of Internal Medicine Teikyo University School of Medicine Tokyo Japan; ^4^ Department of Thoracic Oncology Kanagawa Cancer Center Yokohama Japan; ^5^ Department of Medical Oncology Oita University Faculty of Medicine Yufu Japan; ^6^ Department of Respiratory Medicine Japanese Red Cross Okayama Hospital Okayama Japan; ^7^ Department of Internal Medicine Niigata Cancer Center Hospital Niigata Japan; ^8^ Department of Respiratory Medicine Nagasaki University Graduate School of Biomedical Sciences Nagasaki Japan; ^9^ Department of Respiratory Medicine, Ibaraki Prefectural Central Hospital Ibaraki Cancer Center Kasama Japan; ^10^ Division of Respiratory Medicine Gunma Prefectural Cancer Center Ota Japan; ^11^ Department of Respiratory Medicine, International Medical Center Saitama Medical University Hidaka Japan; ^12^ Department of Biostatistics, Clinical Research Center Shizuoka Cancer Center Sunto Japan; ^13^ Collaborative Research Laboratory St. Luke's International University and Hospital Tokyo Japan; ^14^ Department of Respiratory Medicine and Medical Oncology Yokohama Municipal Citizen's Hospital Yokohama Japan

**Keywords:** advanced, non‐squamous, non‐small cell lung cancer, bevacizumab maintenance therapy, cisplatin, pemetrexed, randomized, Phase II study

## Abstract

**Introduction:**

Cisplatin plus pemetrexed followed by pemetrexed is an efficacious platinum combination regimen for advanced non‐squamous, non‐small cell lung cancer (NSCLC). Data regarding the addition of bevacizumab, especially in maintenance treatment, are insufficient.

**Methods:**

Eligibility criteria included: no prior chemotherapy; advanced, non‐squamous, NSCLC; performance status ≤1; and epidermal growth factor receptor mutation‐negative. Patients (*N* = 108) received induction chemotherapy with cisplatin, pemetrexed, and bevacizumab every 3 weeks for four cycles, and tumor response was needed to confirm four‐week response duration. Patients with at least stable disease were randomized to pemetrexed/bevacizumab or pemetrexed alone. The primary endpoint was progression‐free survival (PFS) after induction chemotherapy. Myeloid‐derived suppressor cell (MDSC) counts of peripheral blood samples were also analyzed.

**Results:**

Thirty‐five patients each were randomized to the pemetrexed/bevacizumab group and the pemetrexed alone group. PFS was significantly better in the pemetrexed/bevacizumab group than in the pemetrexed alone group (7.0 vs. 5.4 months, hazard ratio: 0.56 [0.34–0.93], log‐rank *p* = 0.023). In patients with partial response to induction therapy, median overall survival was 23.3 months in the pemetrexed alone group and 29.6 months in the pemetrexed/bevacizumab group (log‐rank *p* = 0.077). Pretreatment monocytic (M)‐MDSC counts tended to be greater in the pemetrexed/bevacizumab group with poor PFS than in those with good PFS (*p* = 0.0724).

**Conclusions:**

Addition of bevacizumab to pemetrexed as maintenance therapy prolonged PFS in patients with untreated, advanced, non‐squamous NSCLC. Furthermore, an early response to induction therapy and pretreatment M‐MDSC counts may be related to the survival benefit of the addition of bevacizumab to the combination of cisplatin and pemetrexed.

## 1 INTRODUCTION

Non‐small cell lung cancer (NSCLC) has been one of the most common causes of cancer‐related death.^1^, ^2^ Platinum‐based chemotherapy regimens have been studied as first‐line therapy for advanced NSCLC. Meta‐analyses of clinical studies and randomized, clinical studies have shown the survival benefits of platinum‐based chemotherapy for patients with advanced NSCLC.^3^, ^4^, ^5^

NSCLC accounts for 80%–90% of all lung cancers, and non‐squamous cell lung cancer accounts for 70%–80% of all NSCLC cases.^6^, ^7^, ^8^ first‐line platinum chemotherapy regimens for advanced NSCLC have been selected by histology.^8^, ^9^ A randomized, Phase III study showed the significant overall survival benefit of a cisplatin and pemetrexed regimen compared with that of a cisplatin and gemcitabine regimen in patients with advanced non‐squamous NSCLC.^10^ Therefore, a combination of cisplatin or carboplatin and pemetrexed is the standard first‐line platinum chemotherapy regimen for advanced non‐squamous NSCLC.

Bevacizumab is a monoclonal antibody that targets vascular endothelial growth factor (VEGF).^11^ It inhibits angiogenesis of tumor vessels and inhibits tumor growth.^12^ The addition of bevacizumab to carboplatin plus paclitaxel showed a significant overall survival benefit in the treatment of advanced non‐squamous NSCLC.^13^ On the other hand, the addition of bevacizumab to cisplatin or carboplatin plus pemetrexed improved the response rate,^14^ but there were insufficient data to support the survival benefit of the addition of bevacizumab to cisplatin or carboplatin plus pemetrexed in the treatment of advanced, non‐squamous NSCLC, especially in maintenance treatment.

Recently, immune checkpoint‐inhibitors targeting the programmed cell death 1 (PD‐1)/programmed cell death ligand 1 (PD‐L1) have been shown to improve survival in advanced NSCLC.^15^, ^16^, ^17^, ^18^ The analysis of tumor immunity is important for the treatment of cancer. VEGF increases and activates immunosuppressor cells, such as myeloid‐derived suppressor cells (MDSCs) and regulatory T cells (Tregs), and has an immunosuppressive effect.^19^, ^20^, ^21^ Therefore, bevacizumab with a VEGF inhibitory effect may affect tumor immunity.

Thus, a randomized, Phase II study comparing maintenance therapies of pemetrexed plus bevacizumab versus pemetrexed alone after treatment with cisplatin, pemetrexed, and bevacizumab was conducted. The main objectives of the study were to determine the efficacy of the addition of bevacizumab in maintenance therapy for previously untreated advanced non‐squamous NSCLC. This randomized, Phase II study was reported in accordance with the CONSORT (Consolidated Standards of Reporting Trials) Statement. In addition, this study was conceived and initiated before the widespread use of immune checkpoint inhibitors as part of first‐line treatment. The numbers of immunocompetent cells in peripheral blood were analyzed in some patients.

## 2 PATIENTS AND METHODS

### 2.1 Patient eligibility

Patients with histologically or cytologically confirmed non‐squamous NSCLC with measurable disease were enrolled. Other eligibility criteria included: Stage IIIB (without indications for radical chest radiation therapy), Stage IV, or postoperative recurrence; age ≥20 and <75 years; Eastern Cooperative Oncology Group (ECOG) performance status (PS) 0 or 1; no prior chemotherapy, no palliative radiotherapy; epidermal growth factor receptor (EGFR) mutation‐negative; adequate hematopoietic function (leukocyte count ≥4000/μL, neutrophil count ≥2000/μL, hemoglobin ≥9.0 g/dL, platelet count ≥10.0 × 10^4^/μL), hepatic function (total serum bilirubin level ≤1.5 mg/dL, and aspartate aminotransferase (AST) and alanine aminotransferase (ALT) levels ≤100 IU/L), and renal function (serum creatinine level ≤1.2 mg/dL; creatinine clearance ≥60 mL/min); proteinuria ≤1+; percutaneous oxygen saturation (SpO_2_) ≥ 90%; life expectancy greater than 12 weeks; and no co‐existing severe medical problems. Key exclusion criteria included interstitial pneumonia, symptomatic brain metastasis, uncontrolled hypertension, active hemoptysis, active thrombosis, or embolism.

### 2.2 Study design and treatment

Patients received induction chemotherapy of cisplatin (75 mg/m^2^, Day 1), pemetrexed (500 mg/m^2^, Day 1), and bevacizumab (15 mg/kg, Day 1) every 3 weeks for four cycles. The dosage of bevacizumab that is covered by Japan Insurance is 15 mg/kg. Patients with complete response (CR), partial response (PR), and stable disease (SD) to induction therapy were randomized (1:1) to receive pemetrexed plus bevacizumab or pemetrexed alone until progressive disease (PD) or intolerable adverse events or patients' refusal.

The criteria to start maintenance therapy included: PS 0 or 1; neutrophil count ≥1500/μL; hemoglobin ≥8.0 g/dL; platelet count ≥7.5 × 10^4^/μL; total serum bilirubin ≤2.0 mg/dL and AST and ALT ≤100 IU/L; serum creatinine ≤1.5 mg/dL; proteinuria ≤1+; SpO_2_ ≥ 90%; and no co‐existing severe medical problems.

Administration of maintenance therapies was suspended if any of the following occurred: neutrophil count <1500/μL; platelet count <75,000/μL; hemoglobin <8.0 g/dL; total serum bilirubin >2.0 mg/dL; AST and ALT >100 IU/L; serum creatinine >1.5 mg/dL; or Grade 3 non‐hematological toxicities. Administration of only bevacizumab was suspended if there was active hemoptysis or proteinuria ≥2+. When these conditions improved, maintenance therapies were resumed.

This randomized, Phase II study was registered with the UMIN Clinical Trials Registry (number UMIN000010681). The present study protocol was approved by the ethics committees of Tochigi Cancer Center and all participating centers. The present study was conducted in accordance with the Declaration of Helsinki. All patients provided their written, informed consent according to the protocol before study entry. Patients were recruited at 19 investigational sites of the Thoracic Oncology Research Group (TORG) in Japan.

### 2.3 Assessment of treatment

Before commencement of therapy, a complete medical history, physical examination, and resting 12‐lead electrocardiogram were performed. Tumor staging was determined by physical examination, routine chest radiography, computed tomography (CT) of the chest and abdomen, bone scintigraphy, or positron emission tomography (PET), and magnetic resonance imaging (MRI) of the head. Staging was performed according to the 7th edition of the tumor, node, metastasis (TNM) system. A complete blood count including differential leukocyte count, urinalysis, and biochemical analyses were performed two or three times per month during induction therapy and once per course during maintenance therapy. Physical examinations and adverse event assessments according to the Common Toxicity Criteria of Adverse Events, version 4.0 were evaluated for each course of therapy. Tumor efficacy was evaluated radiologically using the Response Evaluation Criteria In Solid Tumors version 1.1. Chest X‐rays were performed at least twice per course during induction therapy and once per course during maintenance therapy.

After the second cycle of induction chemotherapy with cisplatin, pemetrexed, and bevacizumab, CT of the chest and abdomen was performed. After the fourth cycle of induction chemotherapy, CT of the chest and abdomen and MRI of the head were performed between Day 15 and Day 29 after the fourth cycle of induction chemotherapy. In induction therapy, tumor response of CR and PR was needed to confirm the four‐week response duration in this study. After the tumor response evaluation of induction therapies, patients with CR, PR, and SD following induction therapy were randomized (1:1) to receive maintenance therapy with pemetrexed plus bevacizumab or pemetrexed alone. The maintenance therapy started between 3 weeks and 8 weeks after the fourth cycle of induction therapy. During maintenance therapy, CT of the chest and abdomen was performed every 6 weeks, and tumor efficacy was evaluated. All CT and MRI examinations were diagnosed by independent radiologists.

### 2.4 Statistical analysis

The primary endpoint of this study was progression‐free survival (PFS) from randomization. Secondary endpoints were the objective response rate (ORR), overall survival (OS) from randomization, and adverse events. The sample size was calculated with a one‐sided significance level of 0.05 and 80% statistical power using the case number design of Lachin and Foulkes.^22^ The threshold value of median PFS of the pemetrexed alone group (Pgroup) was predicted to be 4.1 months, and the expected value of the median PFS of the pemetrexed plus bevacizumab group (PBgroup) was predicted to be 7.4 months. The threshold value of the median PFS was obtained from the PARAMOUNT study,^23^ and the expected value of the median PFS was obtained from the AVAPERL study.^14^ The sample size of 74 patients, which was the number of randomized patients after induction therapy, was estimated to achieve the desired statistical power. Events in 72 of 74 patients were needed to estimate the primary endpoint. If the disease control rate of induction therapy was 65%, a sample size of 113 patients was needed for starting induction therapy.

All data of this study were maintained in the TORG coordinating office, which was independent of the attending physicians and institutions. All data maintained in the TORG coordinating office were not available to attending physicians. The entries of all patients were conducted by physicians and assistants at each institution. The random allocation was stratified by response to induction therapy (SD, PR, and CR), pathology (adenocarcinoma vs. non‐adenocarcinoma), PS (0 vs. 1), and institution. The minimization method was applied to balance the distribution of prognostic factors between the pemetrexed group and the pemetrexed plus bevacizumab group. Zelen's method was used to balance the number of patients allocated to the two groups.^24^ The rules of random allocation were not available to attending physicians during this study.

Although this study proceeded with the above settings, the combination of immune checkpoint‐inhibitor and chemotherapy has been the standard first‐line therapy for advanced NSCLC since January 2019. It was expected that it would be difficult to collect new cases after 2019 in Japan; therefore, the sample size was re‐calculated with a one‐sided significance level of 0.10 and statistical power of 80% at December 2018. In this setting, a sample size of 54 patients was estimated to achieve the desired statistical power, and events in 52 of 54 patients were needed to estimate the primary endpoint. In addition, 66 patients were randomized at December 2018. Due to these circumstances, case entry was conducted until March 2019 without opening the data. When events occurred in 52 patients, all data were analyzed.

### 2.5 Analysis of circulating immunocytes of peripheral blood

Circulating immunocytes, such as MDSCs, cluster of differentiation (CD) 4 T‐cells, CD8 T‐cells, and Tregs, were analyzed. To analyze circulating immunocytes, peripheral blood samples of patients were collected at four points (before and after induction chemotherapy, 4–8 weeks after the start of maintenance treatment, and after maintenance treatment). The peripheral blood samples of patients were centrifuged at 3000 rpm, and peripheral blood mononuclear cells (PBMCs) were separated by density gradient centrifugation within 6 hours of blood collection. The collected PBMCs were stained by antibodies for surface markers.

The percentage of each subset of PBMCs was counted by flow cytometry. The number of each subset of PBMCs was calculated by multiplying the percentage of each subset of PBMCs by the total number of granulocytes. MDSCs are commonly defined as a CD11b^+^CD33^+^HLA‐DR^−/low^ subset of mononucleated cells. There are two subsets of MDSCs, defined as granulocytic‐MDSC (G‐MDSCs) and monocytic‐MDSCs (M‐MDSCs). M‐MDSCs have been identified based on (CD3/CD16/CD19/CD20/CD56)^−^CD14^+^CD33^+^CD11b^+^HLA‐DR^low/−^, and G‐MDSCs have been identified based on CD33^dim^CD15^+^CD66^+^CD11b^+^. Tregs were defined as a CD4^+^CD25^+^Foxp3^+^ subset of lymphocytes. Significant differences in measured values at each sampling point were examined by the unpaired *t‐*test.

## 3 RESULTS

### 3.1 Patients' characteristics and treatment administration

**FIGURE 1:**
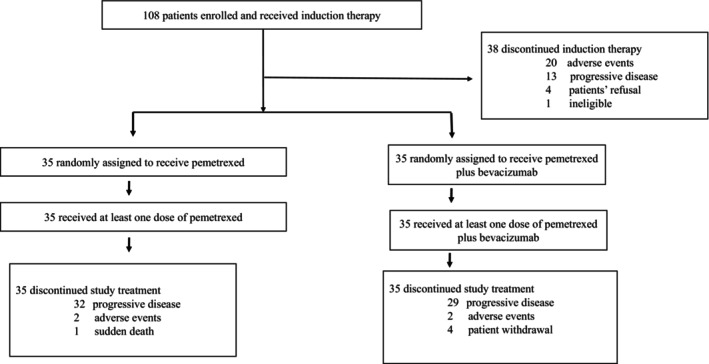
Trial profile.

A total of 108 patients were entered into this study between June 2013 and March 2019. Patients were recruited at 19 investigational sites of TORG in Japan. The patients' characteristics are listed in Table 1. The baseline characteristics of all randomized patients were well balanced. All 108 patients were eligible and received induction chemotherapy. No patients had severe complications. The trial profile is shown in Figure 1.

**TABLE 1:** Patients' background characteristics.

	No. of patients		
	Induction therapy	Maintenance therapy	
		Pemetrexed	Pemetrexed + Bevacizumab
Age (years)			
Median	64	64	63
Range	31–74	31–74	31–74
Sex			
Male	78	24	27
Female	30	11	8
Performance Status			
0	49	16	19
1	59	19	16
Stage			
IIIB	12	6	4
IV	95	28	31
Postoperative recurrence	1	1	0
Pathology			
Adenocarcinoma	103	33	35
Mucoepidermoid	1	1	0
Large cell carcinoma	1	0	0
NSCLC, NOS	3	1	0
Response of induction therapy			
PR	43	20	16
SD	48	15	19

Abbreviations: NSCLC, non‐small cell lung cancer; NOS, not otherwise specified; PR, partial response; SD, stable disease.

The range of maintenance treatment cycles was 1–24 cycles in the Pgroup and 1–42 cycles in the PBgroup. The median number of treatment cycles of maintenance therapy was 7 in the Pgroup and 8 in the PBgroup. The median and mean relative dose intensities of pemetrexed were 85.7% and 87.3% in the PBgroup and 98.1% and 95% in the Pgroup. The median and mean relative dose intensities of bevacizumab were 85.7% and 84.6% in the PBgroup, respectively.

### 3.2 Antitumor activity

Of the 108 eligible patients who received induction chemotherapy, 101 were assessable for response to chemotherapy. Seven patients could not be assessed for response because of discontinuation during induction therapy. The reasons for discontinuation during induction therapy were: patients' refusal, four patients; adverse events, three patients; and ineligible, one patient. None of the patients showed CR, 43 (40.2%) showed PR, 48 (44.9%) showed SD, and nine (8.4%) showed PD. CR and PR were needed to confirm 4 week response duration. All patients who had experienced PR after two cycles of induction therapy maintained the response until the end of the induction therapy. Finally, the overall response rate and disease control rate to chemotherapy were 40.2% (95% confidence interval, 30.8%–50.1%) and 85.0% (95% confidence interval, 76.9%–91.2%), respectively.

**FIGURE 2:**
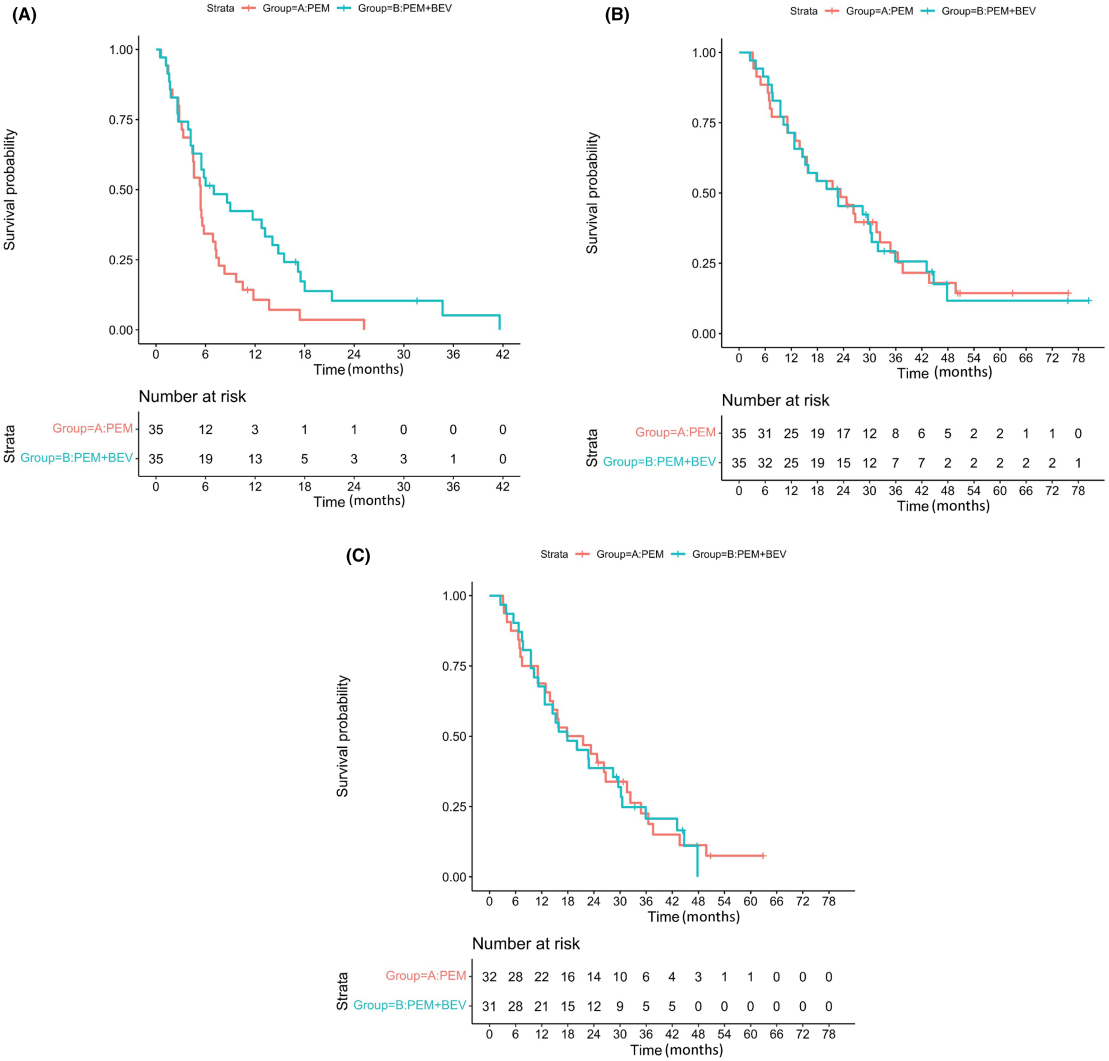
(A) Progression‐free survival from randomization. (B) Overall survival from randomization in an intent to treat analysis. (C) Overall survival from randomization excluded for seven ALK‐positive patients.

PFS of the two groups is shown in Figure 2; 36 of 43 patients who showed PR and 34 of 48 patients who showed SD were randomized after induction therapy, with 35 patients randomized to the Pgroup and 35 patients randomized to the PBgroup. The median PFS was 5.4 (95% confidence interval, 4.4–7.2) months in the Pgroup and 7.0 (95% confidence interval, 4.5–14.8) months in the PBgroup (hazard ratio (HR): 0.56 [0.34–0.93], log‐rank *p =* 0.023). The 1 year PFS rates of the PBgroup and the Pgroup were 37.1% and 8.6%, respectively. The 2 year PFS rates of the PBgroup and the Pgroup were 8.6% and 2.9%, respectively. Addition of bevacizumab to pemetrexed in maintenance therapy was shown to significantly prolong PFS.

OS of the two groups is shown in Figure 2B. Median OS was 23.3 (95% confidence interval, 14.6–34.8) months in the Pgroup and 22.7 (95% confidence interval, 14.5–31.9) months in the PBgroup (HR: 1.03 [95% confidence interval, 0.61–1.73], log‐rank *p* = 0.925). Seven patients were found to have driver mutations other than EGFR mutation after treatment in this clinical study. All seven of these patients were anaplastic lymphoma kinase (ALK) rearrangement‐positive. Three of the seven patients were randomized to the Pgroup, and four of seven patients were randomized to the PBgroup. OS of the two groups except for patients who were ALK rearrangement‐positive is shown in Figure 2C. Median OS was 19.7 (95% confidence interval, 13.9–32.4) months in the Pgroup and 17.9 (95% confidence interval, 12.7–30.5) months in the PBgroup (HR: 1.06 [95% confidence interval, 0.62–1.81], log‐rank *p* = 0.833).

**FIGURE 3:**
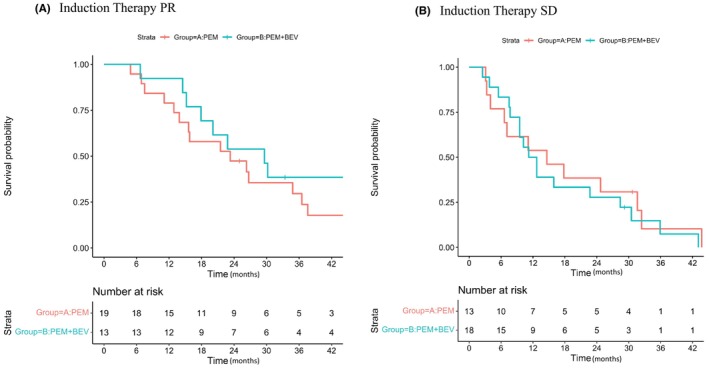
Overall survival from randomization: response to induction chemotherapy.

The OS of the two groups, except for patients who were ALK rearrangement‐positive, separated by their response to induction therapy, such as PR or SD, are shown in Figure 3. In the PR group, the median OS was 23.3 (95% confidence interval, 15.6–37.6) months in the Pgroup and 29.6 (95% confidence interval, 17.9 – NA) months in the PBgroup (HR: 0.50 [95% confidence interval, 0.23–1.08], log‐rank *p* = 0.077). In the SD group, the median OS was 14.6 (95% confidence interval, 6.6 – NA) months in the Pgroup and 12.0 (95% confidence interval, 9.5–30.5) months in the PBgroup (HR: 0.72 [95% confidence interval, 0.34–1.51], log‐rank *p* = 0.377). In the PR group, the median OS tended to be longer in the PBgroup than in the Pgroup. In the SD group, the median OS tended to be slightly shorter in the PBgroup than in the Pgroup.

### 3.3 Adverse events

Grade 3 and 4 hematological adverse events in all cycles of maintenance therapy of all 70 patients are listed in Table 2a. Grade 3 and 4 non‐hematological adverse events except for alopecia in all cycles of maintenance therapy are listed in Table 2b. There were no Grade 4 non‐hematological adverse events. A 72‐year‐old woman in the Pgroup died suddenly after 10 cycles of maintenance therapy. She had no severe adverse events, and the patient's consent for autopsy was not obtained. Therefore, the patient's cause of death was not clear, but coronary artery disease was suspected.

**TABLE 2:** Adverse events (Grade 3 or 4) during maintenance therapy.

	Grade	No. of patients			
		Pemetrexed		Pemetrexed + Bevacizumab	
a. Hematological adverse events					
Leukopenia	3	0		3	≥Grade 3
	4	0		0	8.6%
Neutropenia	3	2	≥Grade 3	2	≥Grade 3
	4	0	5.7%	1	8.6%
Anemia	3	1	≥Grade 3	1	≥Grade 3
	4	0	2.9%	0	2.9%
Thrombocytopenia	3	0		1	≥Grade 3
	4	0		0	2.9%
Febrile neutropenia	3	0	≥Grade 3	1	≥Grade 3
	4	0	0%	0	2.9%
b. Non‐hematological adverse events.					
Anorexia	3	1	≥Grade 3	0	
	4	0	2.9%	0	
Nausea	3	1	≥Grade 3	0	
	4	0	2.9%	0	
Hypoalbuminemia	3	0		1	≥Grade 3
	4	0		0	2.9%
AST/ALT increased	3	0		1	≥Grade 3
	4	0		0	2.9%
Pneumonia	3	0		2	≥Grade 3
	4	0		0	5.7%
Hyponatremia	3	0		1	≥Grade 3
	4	0		0	2.9%
Hyperkalemia	3	1	≥Grade 3	0	
	4	0	2.9%	0	
Hypertension	3	1	≥Grade 3	3	≥Grade 3
	4	0	2.9%	0	8.6%
Edema limbs	3	0		2	≥Grade 3
	4	0		0	5.7%
Hearing impaired	3	1	≥Grade 3	0	
	4	0	2.9%	0	

Abbreviation: AST, aspartate aminotransferase; ALT, alanine aminotransferase.

Proteinuria, hemorrhage from some organs, and hypertension are generally observed in patients who undergo bevacizumab. The number of patients who experienced Grade 3 hypertension was shown in Table 2b. In the Pgroup, Grade 1 and 2 hypertension occurred in 8 and 7 patients, each. In the PBgroup, Grade 1 and 2 hypertension occurred in 13 and 6 patients, each. There were no Grade 3 and 4 proteinuria and hemorrhage. In the Pgroup, Grade 1 and 2 proteinuria occurred in 3 and 2 patients, each. Grade 1 and 2 hemorrhage occurred in 4 and 0 patients, each. In the PBgroup, Grade 1 and 2 proteinuria occurred in 6 and 10 patients, each. Grade 1 and 2 hemorrhage occurred in 5 and one patients, each.

### 3.4 Analysis of circulating immunocytes of peripheral blood

**FIGURE 4:**
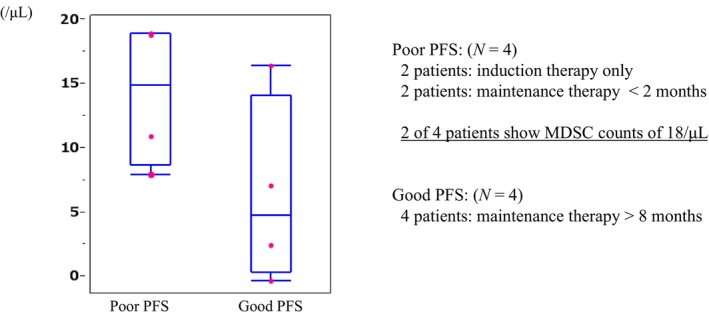
Pretreatment monocytic‐myeloid derived suppressor cell (M‐MDSC) counts of patients in the pemetrexed plus bevacizumab group by progression‐free survival (PFS). Counts tend to be greater in patients with poor PFS than in those with good PFS (*p* = 0.0724).

Circulating immunocytes, such as MDSCs, CD 4 T‐cells, CD8 T‐cells, and Tregs, were analyzed. To analyze circulating immunocytes, peripheral blood samples were collected in ten patients. Eight of the ten patients were from the PBgroup and two of the ten patients were from the Pgroup. The eight patients in the PBgroup were divided into two groups of four each. One group was the poor PFS group, and the other group was the good PFS group. In the poor PFS group, two patients had received induction therapy only, and two patients had received less than two cycles of maintenance therapy consisting of pemetrexed and bevacizumab. The reason for discontinuation of therapy in these four patients was PD. In the good PFS group, all four patients had received induction therapy and more than eight cycles of maintenance therapy, also consisting of pemetrexed and bevacizumab. Pretreatment M‐MDSC counts of peripheral blood of the patients are shown in Figure 4. Pretreatment M‐MDSC counts tended to be higher in the poor PFS group than in the good PFS group (*p* = 0.0724). G‐MDSC counts, Treg counts, and posttreatment M‐MDSC counts did not show differentiation between the poor PFS group and the good PFS group (data not shown).

## 4 DISCUSSION

A randomized, Phase II study of pemetrexed plus bevacizumab versus pemetrexed alone after treatment with cisplatin, pemetrexed, and bevacizumab in untreated advanced non‐squamous, NSCLC was conducted. To the best of our knowledge, this is the first study to demonstrate the significant progression free survival benefit of adding bevacizumab in maintenance therapy after induction therapy with cisplatin, pemetrexed, and bevacizumab for non‐squamous NSCLC. In addition, although this was a small‐sample analysis, this is the first study to have assessed immune‐suppressive cells of human venous blood samples in advanced NSCLC patients treated with chemotherapy including a VEGF inhibitor.

This study showed that the addition of bevacizumab to pemetrexed in maintenance therapy prolonged PFS significantly. In the PARAMOUNT study, which was a Phase III, randomized study comparing pemetrexed with placebo in maintenance therapy, the median PFS of maintenance therapy with pemetrexed alone was 4.1 months.^23^ In the AVAPERL study, which was a Phase III, randomized study comparing pemetrexed plus bevacizumab with bevacizumab in maintenance therapy, the median PFS of maintenance therapy with pemetrexed plus bevacizumab was 7.4 months.^14^ In the present study, the median PFS was 5.4 months in the pemetrexed alone group and 7.0 months in the pemetrexed and bevacizumab group. These data were similar and reproducible to the AVAPERL study and the PARAMOUNT study. Therefore, the significant survival benefit of adding bevacizumab in maintenance therapy in this study was further confirmed. On the other hand, in the COMPASS study, which was a Phase III, randomized study comparing pemetrexed plus bevacizumab with bevacizumab in maintenance therapy after the induction of carboplatin, pemetrexed, and bevacizumab, the median PFS of maintenance therapy with pemetrexed plus bevacizumab were 5.7 months.^25^ This data might suggest that carboplatin and cisplatin are equally effective.

Recently, the addition of immune checkpoint‐inhibitors to platinum‐based chemotherapy showed significant survival benefits over chemotherapy alone in advanced, non‐squamous NSCLC.^17^ The combination therapy of immune checkpoint‐inhibitors and platinum‐based chemotherapy is the standard chemotherapy in advanced, non‐squamous NSCLC. However, lung cancer patients with immune complications cannot be treated with immune checkpoint‐inhibitors. In addition, the survival benefit of immune checkpoint‐inhibitors alone has not been sufficient in NSCLC patients with PD‐L1 expression of less than 50%. Therefore, increasing the effectiveness of platinum‐based chemotherapy has remained important. Furthermore, the combination therapy of immune checkpoint‐inhibitors, platinum‐based chemotherapy, and bevacizumab is one of the standard chemotherapies in advanced, non‐squamous NSCLC. However, the survival benefits of the addition of bevacizumab to immune checkpoint‐inhibitors and platinum‐based chemotherapy have not been clear.^26^ The results of the present study support the survival benefits of the addition of bevacizumab to platinum‐based chemotherapy, and they may support the survival benefits of the addition of bevacizumab to the combination of immune checkpoint‐inhibitors and platinum‐based chemotherapy.

Some previous studies could not show sufficient data to support the OS benefit of the addition of bevacizumab to platinum‐based chemotherapy in the treatment of advanced, non‐squamous NSCLC. However, we have often experienced cases in which platinum‐based chemotherapy with bevacizumab is highly effective. The present study has limitations in assessing OS. This is because OS is not included in the primary endpoint and the number of cases is small to assess OS. In the present study, OS tended to be similar in the Pgroup and the PBgroup. However, when the response to induction therapy was PR, the median OS tended to be longer in the PBgroup than in the Pgroup. When the response to induction therapy was SD, the median OS tended to be similar in the PBgroup and the Pgroup. In the present study, the evaluation of PR to induction therapy needed to confirm a response duration of 4 weeks. Therefore, the tumor reduced clearly within two cycles of induction therapy in patients with PR. Many previous studies of advanced NSCLC have not shown that the addition of bevacizumab could prolong OS significantly.^27^, ^28^ On the other hand, some studies have shown that the addition of bevacizumab could prolong OS significantly.^16^, ^29^ These results may be because the proportion of patients who have overall survival benefits with bevacizumab is not very high. It would be useful if predictors of the effect of bevacizumab were known, but they are not yet known. PR within two cycles of induction therapy with cisplatin, pemetrexed, and bevacizumab may be a predictor of the OS benefits of the addition of bevacizumab. Therefore, based on this study, about 40% of non‐squamous NSCLC patients who have early PR may have OS benefits with bevacizumab. Biomarkers that can identify these patients are needed. In addition, bevacizumab in the maintenance phase for patients who experienced SD after the induction triplet regimen may not be necessary.

Recently, VEGF has been found to increase and activate immunosuppressor cells, such as MDSCs and Tregs, and their effects.^19^, ^20^, ^21^ Therefore, bevacizumab with its VEGF inhibitory effect may affect tumor immunity, and immunosuppressor cells may be the predictors of the effect of bevacizumab. In the present study, peripheral blood samples were collected at four time points per patient. Although the analyzed data were from only eight patients, pretreatment M‐MDSC counts in the poor PFS group tended to be greater than those in the good PFS group. The fact that pretreatment M‐MDSC counts in peripheral blood tended to be greater in poor PFS group than in good PFS group might suggest that the effect of bevacizumab was limited, and if the M‐MDSC level was high, bevacizumab might not be able to suppress the immunosuppressive effects of M‐MDSC completely. In the present study, data on M‐MDSC counts in Pgroup were available for only two subjects, so it was not possible to compare them with those in PBgroup. Therefore, the correlation between M‐MDSC counts and effects of bevacizumab could not be examined. However, this result demonstrates that pretreatment M‐MDSC counts may predict the effect of the combination therapy of cisplatin, pemetrexed, and bevacizumab. In addition, this result demonstrates that the possibility of the effects of bevacizumab on immune function. Although further studies will be needed to confirm this prediction, the addition of bevacizumab to immune checkpoint‐inhibitors may enhance the survival benefit in advanced non‐squamous NSCLC.

In conclusion, a randomized, Phase II study comparing maintenance therapies of pemetrexed plus bevacizumab versus pemetrexed alone after treatment with cisplatin, pemetrexed, and bevacizumab was conducted. The addition of bevacizumab to pemetrexed in maintenance therapy after treatment with cisplatin, pemetrexed, and bevacizumab is effective with tolerable toxicities in untreated, advanced, non‐squamous NSCLC. The potential effects of bevacizumab on immune function were also suggested. Therefore, bevacizumab should be considered in combination with cisplatin, pemetrexed, and immune checkpoint‐inhibitors. In addition, early PR to induction therapy and pretreatment M‐MDSC counts in peripheral blood may be related to the survival benefit of the addition of bevacizumab to the combination of cisplatin and pemetrexed.

## AUTHOR CONTRIBUTIONS

**Takashi Kasai:** Conceptualization (equal); formal analysis (lead); investigation (lead); methodology (lead); project administration (lead); resources (lead); writing – original draft (lead); writing – review and editing (lead). **Kiyoshi Mori:** Conceptualization (supporting); investigation (supporting); writing – original draft (supporting). **Yoichi Nakamura:** Data curation (supporting). **Nobuhiko Seki:** Data curation (supporting); investigation (supporting). **Yasuko Ichikawa:** Data curation (supporting). **Haruhiro Saito:** Data curation (supporting). **Tetsuro Kondo:** Data curation (supporting). **Kazuo Nishikawa:** Data curation (supporting). **Satoshi Otsu:** Data curation (supporting). **Akihiro Bessho:** Data curation (supporting). **Hiroshi Tanaka:** Data curation (supporting). **Hiroyuki Yamaguchi:** Data curation (supporting). **Takayuki Kaburagi:** Data curation (supporting). **Hisao Imai:** Data curation (supporting). **Keita Mori:** Data curation (supporting). **Junya Ohtake:** Data curation (supporting). **Hiroaki Okamoto:** Conceptualization (supporting); data curation (supporting); funding acquisition (supporting); project administration (supporting).

## FUNDING INFORMATION

This research did not receive any specific grant from funding agencies in the public, commercial, or not‐for‐profit sectors.

## CONFLICT OF INTEREST STATEMENT

The authors have declared no conflicts of interest.

## Data Availability

Data sharing is not applicable to this article as no new data were created or analyzed in this study.
